# TP53 supports basal-like differentiation of mammary epithelial cells by preventing translocation of deltaNp63 into nucleoli

**DOI:** 10.1038/srep04663

**Published:** 2014-04-11

**Authors:** Pauliina M. Munne, Yuexi Gu, Manuela Tumiati, Ping Gao, Sonja Koopal, Sanna Uusivirta, Janet Sawicki, Gong-Hong Wei, Sergey G. Kuznetsov

**Affiliations:** 1 Institute for Molecular Medicine Finland (FIMM), University of Helsinki, PO Box 20, FIN-00014, Helsinki, Finland; 2 Biocenter Oulu and Department of Medical Biochemistry and Molecular Biology, Faculty of Biochemistry and Molecular Medicine, University of Oulu, P.O. Box 5000, Oulu FIN-90014, Finland; 3 Lankenau Institute for Medical Research, 100 Lancaster Avenue, Wynnewood, PA 19096, USA

## Abstract

Multiple observations suggest a cell type-specific role for *TP53* in mammary epithelia. We developed an *in vitro* assay, in which primary mouse mammary epithelial cells (mMECs) progressed from lumenal to basal-like phenotypes based on expression of Krt18 or ΔNp63, respectively. Such transition was markedly delayed in *Trp53^−/−^* mMECs suggesting that Trp53 is required for specification of the basal, but not lumenal cells. Evidence from human basal-like cell lines suggests that TP53 may support the activity of ΔNp63 by preventing its translocation from nucleoplasm into nucleoli. In human lumenal cells, activation of TP53 by inhibiting MDM2 or BRCA1 restored the nucleoplasmic expression of ΔNp63. *Trp53^−/−^* mMECs eventually lost epithelial features resulting in upregulation of MDM2 and translocation of ΔNp63 into nucleoli. We propose that *TP63* may contribute to *TP53*-mediated oncogenic transformation of epithelial cells and shed light on tissue- and cell type-specific biases observed for TP53-related cancers.

T*P53* is a bona fide tumor suppressor somatically mutated in almost half of all human cancers. Its tumor suppressor activity is typically ascribed to its role as a transcription factor regulating expression of genes involved in control of cell cycle, cellular senescence, and apoptosis^1^. Aberrations in such common cellular processes, however, do not explain known p53-associated developmental defects in embryonic tissues of epidermal origin in female mice or a strong tissue-specific bias in the tumor spectrum. *Trp53* knock-out mice mostly develop lymphomas and sarcomas^2^,^3^,^4^, while concurrent mutations in some DNA repair genes may, however, shift the tumor spectrum toward epithelia-derived carcinomas^5^. In addition, while cancer-associated point mutations in the *TP53* gene are equally common in both lumenal and basal-like breast cancers, truncating mutations and large scale deletions in this gene are more prevalent in basal-like breast cancers compared with the lumenal subtypes suggesting that different cell types within mammary epithelia may have different requirements for *TP53*^6^.

Mammary glands are ectodermal organs composed of two major cell types: lumenal cells form the inner layer of the mammary ducts and alveoli, and produce milk as their primary function; basal myoepithelial cells synthesize the basement membrane separating mammary epithelia from surrounding adipose tissues and provide a contractile function for milk secretion. Mammary lumenal cells are commonly characterized by expression of cytokeratins 8 and 18 (KRT8/18), while basal epithelial cells, in contrast, express cytokeratins 5 and 14 (KRT 5/14) and an isoform of TP63 known as ΔNp63^7^. TP63 is a member of the p53 protein family sharing an extensive sequence similarity, oligomerization and DNA binding abilities^8^. The ΔNp63 isoform is transcribed from an alternative promoter located in the third intron and, in contrast to the full-length isoform known as TAp63, lacks the transactivation domain at the amino-terminus^9^. TAp63 transactivates many of the TP53-regulated genes, while ΔNp63 lacks this capacity and is thought to function in a dominant negative fashion^9^. In contrast to *TP53*, *TP63* is rarely mutated in cancers and is known to play essential developmental functions^10^,^11^. TAp63 is almost undetectable in adult tissues except for oocytes and rapidly renewing B-lymphocytes, but induced during wound healing and genotoxic stress while ΔNp63 is widely expressed in the basal layers of multiple epithelial tissues where it plays essential and complex roles in stem cell maintenance and differentiation^12^,^13^,^14^,^15^,^16^. Given such essential roles that *TP63* plays in epidermal tissues, and the presence of multiple TP53 binding sites in both promoters of *TP63* (Supplementary Figure S1), it is possible that *TP63* may serve as a mediator of the cell type-specific effects of *TP53*.

No cell type-restricted abnormalities within mammary epithelia are reported for existing *Trp53*-mutated mouse models except for a delayed involution after pregnancy, which is likely related to its pro-apoptotic function^4^. To reveal possible effects of *Trp53* on the differentiation of lumenal and basal epithelial lineages, we developed an *in vitro* differentiation assay, in which primary mouse mammary epithelial cells (mMECs) are explanted in a plastic dish and their differentiation is monitored using cell type-specific markers over time. Our data demonstrate that *Trp53* is required for differentiation of basal epithelial cells, while having an opposite effect on the lumenal cells. Studies on human mammary epithelial cell lines suggest that in basal epithelial cells, TP53 inhibits expression of the TAp63 isoform, while supporting the activity of ΔNp63. Our experiments indicate that inactivation of ΔNp63 may occur by sequestering the protein in nucleoli. This work suggests that *TP63* may be an essential component of the *TP53*-mediated oncogenic transformation of epidermal cells.

## Results

### Loss of p53 inhibits differentiation of basal-like mammary cells

**Figure 1:**
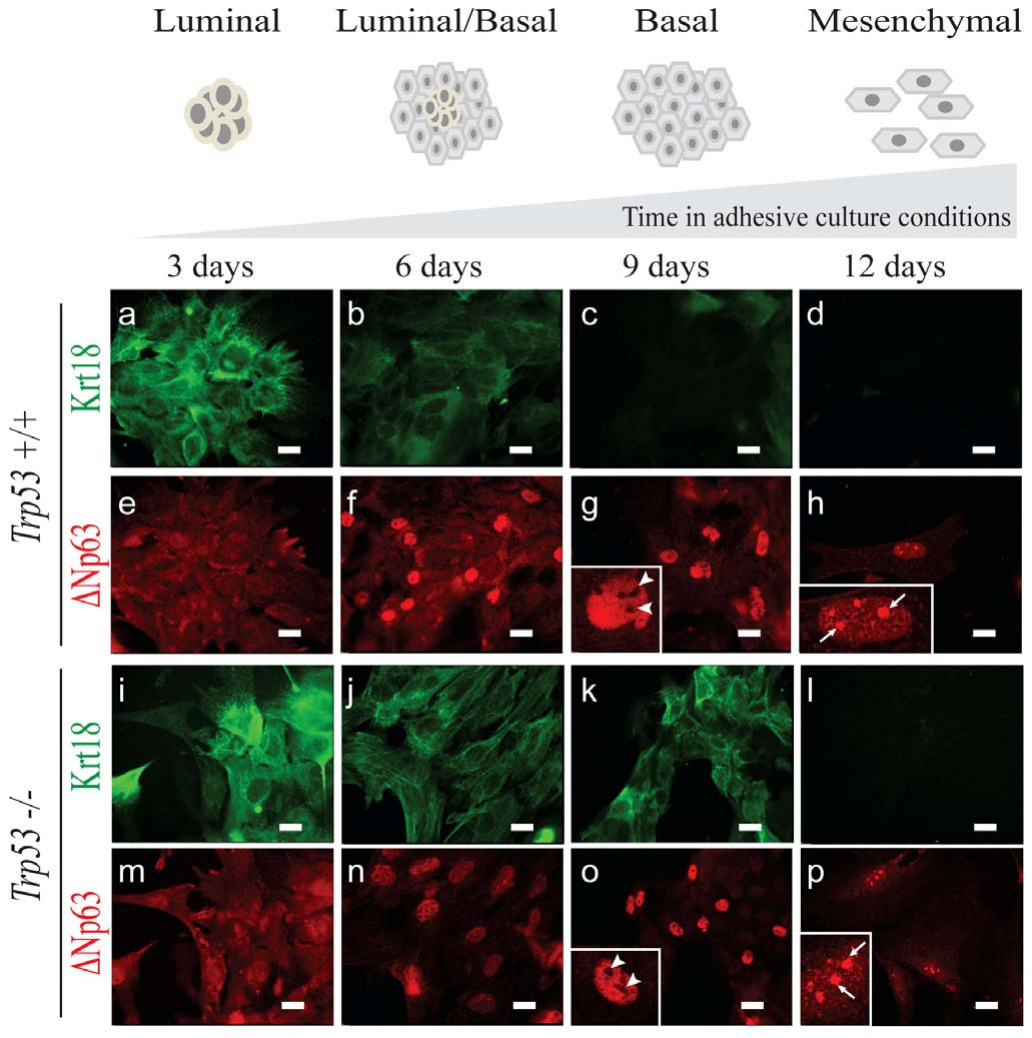
Loss of *Trp53* in mMECs cultured in adhesive conditions delays their transition from a luminal to basal-like phenotype. Freshly isolated *Trp53^+/+^* (a–h) and *Trp53^−/−^* (i–p) primary mMECs were cultured in plastic dishes and sampled every three days. (a–d, i–l) Luminal differentiation was monitored by immunofluorescence staining for cytokeratin 18 (Krt18, green). (e–h, m–p) Basal differentiation was monitored by staining for ΔNp63 (red). Insets in (g, *h, o, and p*) demonstrate selected nuclei at a higher magnification. Arrowheads indicate ΔNp63 excluded from nucleoli. Arrows show ΔNp63 located in nucleoli. Scale bars correspond to 20 μm.

To reveal a role of *Trp53* in the differentiation of lumenal and basal mammary epithelial lineages, we developed an *in vitro* differentiation system, in which freshly isolated mMECs gradually, in a course of 12 days, switched expression of lineage markers from lumenal to basal, eventually losing them altogether (Figure 1). Here, Krt18 detected by immunofluorescence was used as a marker of lumenal (Figure 1a–d, and i–l), and ΔNp63 – as a marker of basal differentiation (Figure 1e–h, and m–p). Most wild type mMECs expressed only the lumenal marker for the first three days in culture (Figure 1a), which became weaker at day 6, and essentially disappeared by day 9 (Figure 1b, c). In contrast, the basal marker ΔNp63 could be reliably detected only after six days in culture (Figure 1e–g). These phenotypic changes were independently confirmed using wild type primary mMECs isolated from reporter mice expressing a red (RFP) and cyan (CFP) fluorescent proteins under a Krt18 or Krt5 promoters serving as a lumenal or basal markers, respectively (Supplementary Figure S2). There, both RFP- and CFP-positive cells could be found during the first 2 days in culture, while only the CFP reporter was evidently expressed in all cells after 5 days, and both disappeared on day 7 in culture (Supplementary Figure S2). Unlike wild type cells, *Trp53^−/−^* mMECs demonstrated a sustained Krt18 expression even after nine days in culture (Figure 1i–k), while ΔNp63 remained weakly expressed at days 3 and 6, becoming stronger only at day 9 (Figure 1m–o). Together, this suggests that Trp53 counteracts the lumenal differentiation and promotes the basal-like lineage.

### EMT correlates with a relocation ofΔNp63 from the nucleoplasm into nucleoli

**Figure 2:**
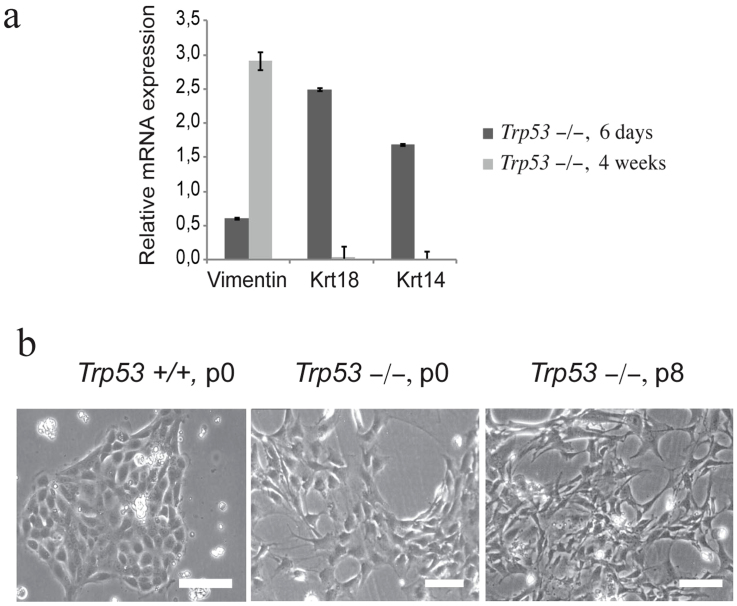
*Trp53^−/−^* mMECs acquire mesenchymal features *in vitro*. (a) Expression of a mesenchymal marker Vimentin increases, while that of epithelial keratins (Krt18 and Krt14) decreases with time, when mMEC cultures at 6 days and 4 weeks were compared using qRT-PCR. The bar plot shows the mRNA expression as a fold change relative to wild type mMECs. (b) Phase contrast images illustrate a spindle-shaped morphology of primary *Trp53^−/−^* mMECs (middle) in contrast to a tight epithelial sheet of wild type mMECs (left). Extensively passaged *Trp53^−/−^* mMECs morphologically resemble fibroblasts (right). Scale bars correspond to 100 μm.

At day 12 both wild type and *Trp53^−/−^* mMECs stained negative for Krt18 (Figure 1d,l). Most wild type mMECs also stopped proliferating around this time and morphologically resembled senescent fibroblasts (data not shown). In contrast, *Trp53^−/−^* mMECs could proliferate indefinitely consistent with a reported ability of a mutant *Trp53* to immortalize primary cells^17^. Extensively cultured *Trp53^−/−^* mMECs lost expression of both lumenal and basal keratins, Krt18 and Krt5, respectively (Figure 2a). They also upregulated a mesenchymal marker Vimentin (Figure 2a), suggesting that mMECs undergo an epithelial-to-mesenchymal transition (EMT) when cultured on plastic. Loss of *Trp53* was reported to promote EMT^18^ and, indeed, even at the earliest passage colonies of *Trp53^−/−^* mMECs were less compact than the wild type counterparts and morphologically resembled fibroblasts or mesenchymal cells rather than polarized cuboidal epithelial cells (Figure 2b). These morphological changes progressed even further when *Trp53^−/−^* mMECs were cultured for eight passages (Figure 2b).

**Figure 3:**
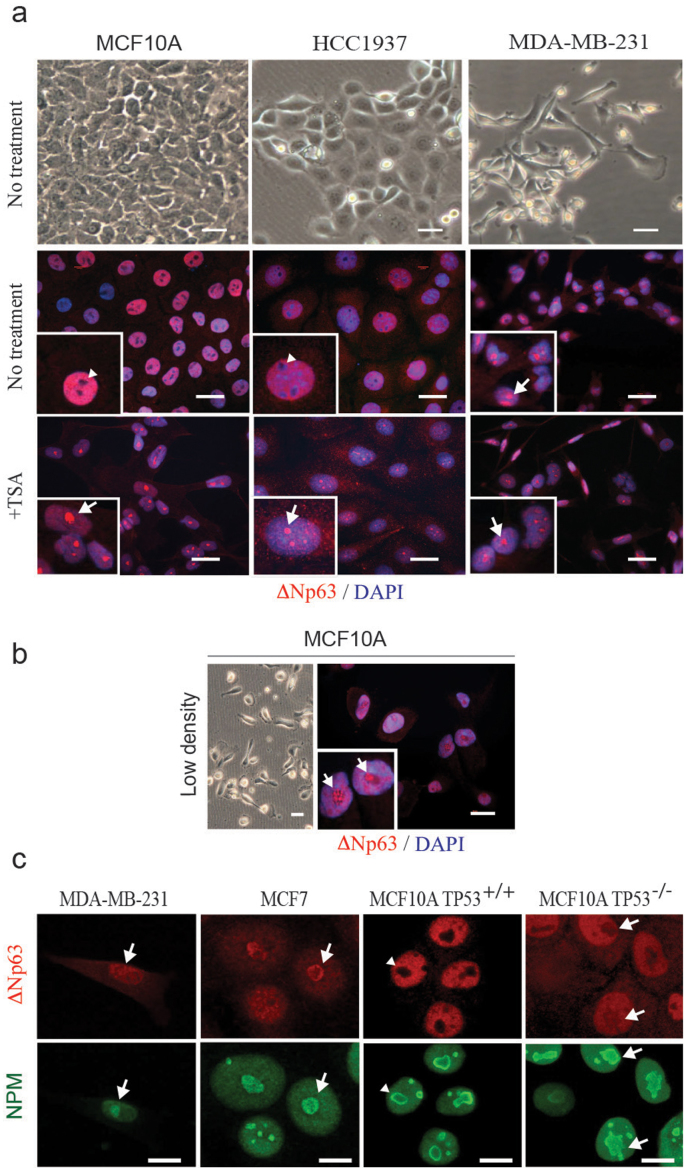
Nucleolar localization of ΔNp63 correlates with EMT and functional inactivation. (a) Phase contrast images (upper row) demonstrate an epithelial cobblestone-like (MCF10A and HCC1937) and mesenchymal (MDA-MB-231) morphology of human basal mammary cell lines. In cobblestone-like cells, ΔNp63 is excluded from nucleoli (arrowheads, second row). In mesenchymal-like cells, ΔNp63 is found in nucleoli (arrow). Functional inactivation of ΔNp63 using HDAC1 inhibitor trichostatin A (+TSA, third row) leads to a relocation of ΔNp63 into nucleoli in all cell lines. (b) MCF10A cells plated at a low density spontaneously undergo EMT and demonstrate a mesenchymal morphology. In these cells ΔNp63 is found predominantly in nucleoli (arrows). Insets demonstrate higher power views of selected cells in corresponding samples. (c) Co-staining of ΔNp63 with a nucleolar marker nucleophosmin (NPM) showing a nucleolar localization of ΔNp63 protein in MDA-MB-231, MCF7 and *TP53^−/−^* MCF10A cells, but not the parental MCF10A cells. Red, ΔNp63; blue, DAPI. Scale bars correspond to 20 μm in (**a**–b), and 10 μm in (c).

This loss of epithelial features in mMECs also affected such an important epithelial regulator as ΔNp63 protein. While in primary mMECs at 9 days in culture ΔNp63 protein was found in the nucleoplasm excluded from nucleoli (Figure 1g, o), its localization switched to the opposite three days later with most of the protein now found in nucleoli (Figure 1h, p). Therefore, we hypothesized that the nucleolar localization of ΔNp63 is related to the EMT process. To test this hypothesis, we stained basal human mammary epithelial cell lines HCC1937 and MCF10A with a cobblestone-like morphology and found the ΔNp63 protein exclusively in the nucleoplasm (Figure 3a, middle row, and Figure 3c). Exclusion of ΔNp63 from nucleoli in MCF10A cells was confirmed by a co-staining with a nucleolar phosphoprotein B23 also known as nucleophosmin (NPM) (Figure 3c). In contrast, in a mesenchymal-like cell line MDA-MB-231, ΔNp63 was not excluded from nucleoli (Figure 3a, c). Furthermore, MCF10A cells were reported to assume a spindle-like morphology when plated at a low density (compare Figure 3a and b)^19^. In such conditions, the ΔNp63 protein was found predominantly in nucleoli (Figure 3b). This suggests that ΔNp63 shuffles between the nucleoplasm and nucleoli in connection with changes between epithelial and mesenchymal appearances.

What is the functional difference between ΔNp63 located in the nucleoplasm or nucleoli? Silencing of ΔNp63 is known to increase migratory properties of cancer cells, while its lower expression level in tumors is associated with an increased expression of EMT-promoting transcription factors Snail and Slug^20^. Therefore, we hypothesized that translocation of the ΔNp63 protein into nucleoli reflects its functional inactivation. To test this, we used trichostatin A (TSA), a potent inhibitor of histone deacetylases 1 and 2 (HDAC1/2), which are important for ΔNp63-mediated transcriptional repression^21^, to inhibit ΔNp63 in epithelial-like MCF10A and HCC1937 cells. Such treatment resulted in a nucleolar translocation of ΔNp63 in these cells (Figure 3a, bottom row). However in mesenchymal-like MDA-MB-231 cells, ΔNp63 remained in nucleoli regardless of the TSA treatment (Figure 3a). These data support the view that the nucleolar ΔNp63 protein is equivalent to its functional inactivation, and promotes the EMT process.

### TP53 and ΔNp63 in basal cells are antagonized by TAp63

**Figure 4:**
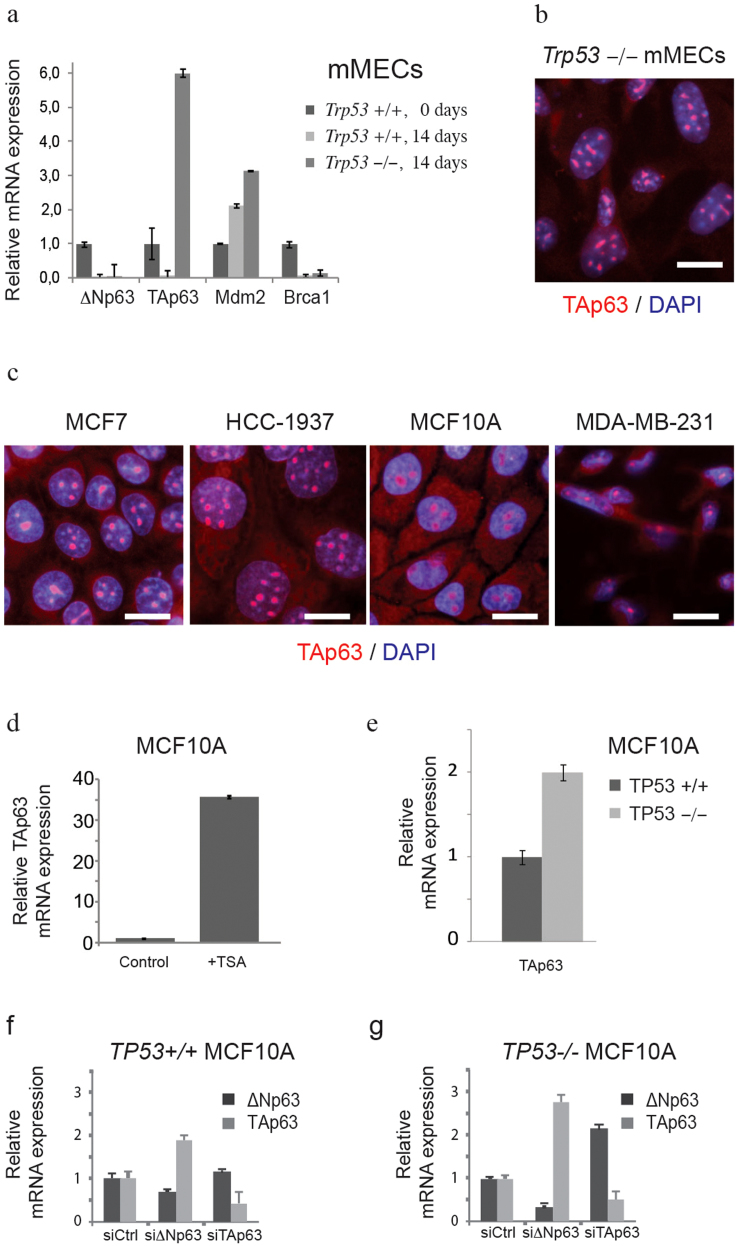
TP53 and ΔNp63 negatively regulate TAp63. (a) mRNA expression was measured using qRT-PCR in *Trp53^−/−^* and wild type mMECs cultured for 14 days. Values represent fold changes relative to wild type mMECs. Notice a sharp increase in TAp63 in *Trp53^−/−^* mMECs. (b) Immunofluorescence staining of immortalized *Trp53^−/−^* mMECs showing a nucleolar localization of TAp63 isoform (red). Nuclei are counterstained with DAPI shown in blue. (c) TAp63 is detected in nucleoli in all tested stable mammary epithelial cell lines. (d) qRT-PCR showing a 36-fold increase in the mRNA level of TAp63 in MCF10A cells after ΔNp63 was inhibited using TSA treatment. (e) qRT-PCR showing a two-fold increase in expression of TAp63 in isogenic *TP53^−/−^* compared with its parental *TP53^+/+^* MCF10A cell line. (f–g) qRT-PCR showing a negative regulation of TAp63 by ΔNp63 in *TP53^+/+^* MCF10A cells (f), and their mutual suppression in *TP53^−/−^* MCF10A cells (g). Scale bars correspond to 20 μm.

As mentioned earlier, ΔNp63 is an alternative product of the *TP63* gene, lacking the transactivation domain. Multiple studies demonstrated an inverse correlation between ΔNp63 and TAp63 isoforms^9^. Having found a negative effect of the *Trp53*-deletion on expression of ΔNp63, we sought to extend our analysis to the TAp63 isoform. Wild type mMECs cultured for 14 days lost expression of both isoforms of *Trp63* (Figure 4a). At the same time the *Trp53^−/−^* mMECs strongly upregulated the TAp63, but not the ΔNp63 isoform (Figure 4a). An isoform-specific antibody staining of immortalized *Trp53^−/−^* mMECs revealed the TAp63 protein located in nucleoli (Figure 4b)^22^. This suggested that a high expression of TAp63 could be directly regulated by Trp53. To address this question, we stained four human mammary cell lines for the TAp63 protein isoform (Figure 4c). Surprisingly, TAp63 protein was expressed in nucleoli in all cell lines without apparent differences between *TP53*-mutated (HCC1937 and MDA-MB-231) and *TP53*-wild type cells (MCF7 and MCF10A), or between basal-like epithelial cells showing either a nucleoplasmic (HCC1937 and MCF10A, Figure 4c) or a nucleolar (MDA-MB-231, Figure 3f) localization of ΔNp63 protein. Nevertheless, inhibition of ΔNp63 in MCF10A cells using the TSA treatment led to a 36-fold increase in the TAp63 expression level supporting the idea that ΔNp63 negatively regulates TAp63 (Figure 4d). In addition, our analysis of isogenic MCF10A cells after a genetic deletion of *TP53* revealed a two-fold increase of TAp63 expression level (Figure 4e). Furthermore, an isoform-specific knockdown of ΔNp63 in *TP53^+/+^* MCF10A cells resulted in a 2-fold increase in TAp63 mRNA, but not *vice versa* (Figure 4f). However, in isogenic *TP53^−/−^* MCF10A cells knockdown of ΔNp63 lead to a 2.5-fold higher TAp63, while knockdown of TAp63 resulted in a 2-fold increase in ΔNp63 (Figure 2g). Taken together, this indicates that expression of TAp63 is controlled by ΔNp63 and TP53. The fact that TAp63 is barely expressed in most adult cell types *in vivo* but readily found in cell lines, supports the view proposed elsewhere that the TAp63 isoform is essential to sustain the self-renewal capacity^23^.

### Activation of TP53 in lumenal cells forces expression of basal markers

**Figure 5:**
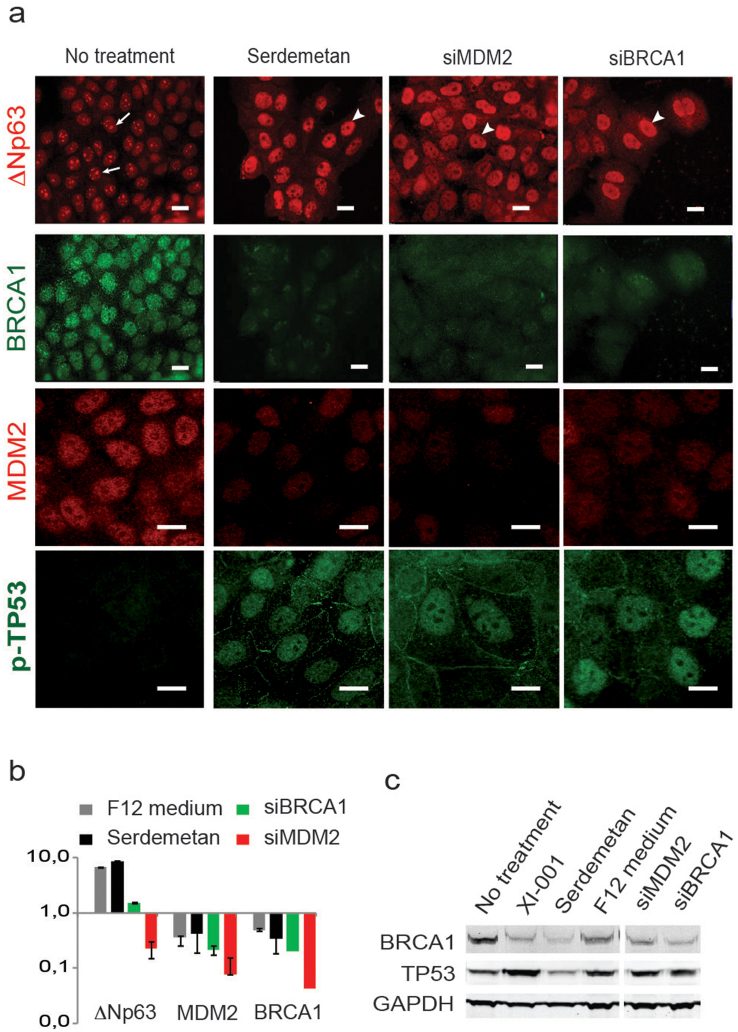
ΔNp63 is suppressed by MDM2 and BRCA1 in lumenal MCF7 cells. (a) Immunofluorescence showing the ΔNp63 protein (top row) in MCF7 cells treated as indicated above. BRCA1 protein (second row) is readily detectable in control cells, but disappears after treatments promoting expression of ΔNp63. MDM2 protein (third row) correlates with the expression of BRCA1. (b) qRT-PCR showing fold change in mRNA levels for ΔNp63, MDM2, and BRCA1 following treatments indicated above. (c) Western blot confirming an effective reduction of BRCA1 protein level and stabilization of the TP53 protein after treatments indicated above. F12, cells were cultured in DMEM/F12 medium instead of the regular DMEM; Serdemetan, cells treated with MDM2 inhibitor as described in Materials and Methods; siBRCA1 and siMDM2, cells treated with siRNAs targeting corresponding genes. Scale bars correspond to 20 µm.

If deletion of *Trp53* were indeed the reason for a delayed switch from lumenal to basal markers observed in primary mMEC cultures, we would expect that an activation of TP53 in lumenal cells would force them to express basal-like markers. TP53 is mainly controlled by E3 ubiquitin ligases MDM2 and MDM4 that bind TP53 and target it for a proteasomal degradation^24^,^25^. Therefore, we pharmacologically inhibited MDM4 using a pseudourea derivative XI-001 in the lumenal breast cancer cell line MCF7 carrying a wild type *TP53* gene and, indeed, observed a strong re-expression of the TP53 protein (Supplementary Figure 3). An inhibition of MDM2 with Serdemetan, a specific compound blocking its E3 ligase activity led to a similar result (Figure 5a). As expected, this treatment increased the expression level of a basal marker ΔNp63 in lumenal MCF7 cells almost 10-fold, which located in the nucleoplasm, but not in nucleoli (Figure 5a, b). The same effect on the ΔNp63 protein was obtained when we knocked-down MDM2 using a specific siRNA (Figure 5a). Interestingly, the level of ΔNp63 mRNA was reduced after the siRNA-mediated knockdown of MDM2 in spite of the visible increase in the corresponding protein (Figure 5a, b). This suggests either that a pharmacological inhibition of MDM2 has a broader specificity than the siRNA or that regulation of ΔNp63 occurs mostly at the post-translational level. Either way, the data confirm that a reactivation of the TP53 pathway in a lumenal cell line can, indeed, lead to expression of basal-specific differentiation markers.

Another way to activate the TP53 pathway is to induce DNA damage checkpoints by inhibiting DNA repair, e.g. via inactivation of BRCA1. BRCA1 is a critical mediator of the homologous recombination pathway of DNA repair. Its germ-line mutations predispose to breast cancers, which tend to have a basal-like phenotype^26^. It is demonstrated, however, that basal *BRCA1*-related breast cancers originate from lumenal progenitor cells^27^. Inhibition of BRCA1 in lumenal MCF7 cells using a specific siRNA resulted in a strongly upregulated basal marker, ΔNp63 protein, both at the mRNA and protein levels (Figure 5a, b, c). This also led to a transcriptional repression of MDM2 (Figure 5b). Equally, inhibition of MDM2 either with Serdemetan or a siRNA suppressed BRCA1 (Figure 5a, b, c) revealing a strong correlation between these two genes.

Somewhat unexpectedly, we found that placing lumenal MCF7 cells into the DMEM/F12 medium, normally used to maintain basal-like MCF10A cells, led to the activation of TP53 protein expression (Supplementary Figure S3). Consistent with the previous observations, this treatment also induced a strong expression of the basal marker ΔNp63 and suppressed BRCA1 and MDM2 (Supplementary Figure 4). When now TP53 was inhibited using a specific siRNA, ΔNp63 protein translocated into nucleoli (Supplementary Figure 4) suggesting its functional inactivation and further supporting a direct link between these genes. In addition, the expression of BRCA1 and MDM2 greatly increased (Supplementary Figure 4). These results establish an association of BRCA1 and MDM2 with the lumenal subtype as opposed to TP53 and ΔNp63 associated with the basal epithelial subtype, and suggest that a switch from the lumenal to basal phenotypes is coordinately regulated by TP53, ΔNp63, MDM2/4, and BRCA1.

### Deletion of TP53 in basal cells induces expression of lumenal markers

**Figure 6:**
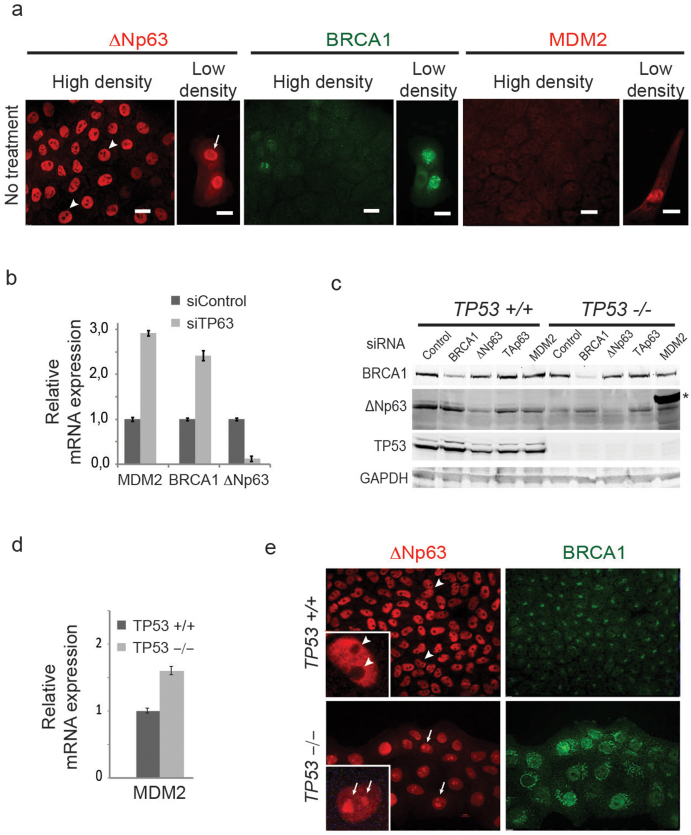
TP53 and ΔNp63 negatively regulate MDM2 and BRCA1 in basal-like cells. (a) Untreated MCF10A cells express high levels of ΔNp63, and low levels of BRCA1 and MDM2. Cells plated at a low density translocate ΔNp63 to nucleoli, and elevate expression of BRCA1, and MDM2. (b) Knockdown of TP63 (siTP63) results in upregulation of BRCA1 and MDM2. (c) Western blot confirming an efficient siRNA-mediated knockdown of BRCA1 and ΔNp63 proteins, and lack of TP53 in *TP53^−/−^* MCF10A cells. Notice a higher level of ΔNp63 protein in BRCA1-depleted TP53-mutant cells. Asterisk indicates a 75 kDa band from a molecular weight marker loaded in the same lane as the siMDM2 sample. (d) TP53-deficiency in MCF10A cells leads to an increase in MDM2 mRNA level as measured by qRT-PCR. (**e**) Immunofluorescence staining of *TP53^−/−^* and its parental isogenic *TP53^+/+^* MCF10A cell lines demonstrates a ubiquitous relocation of ΔNp63 protein from the nucleoplasm in *TP53^+/+^* cells (arrowheads) to nucleoli in *TP53^−/−^* cells (arrows), and upregulation of BRCA1 in *TP53^−/−^* cells. Insets illustrate the differences at a higher magnification. Scale bars correspond to 20 µm.

Non-transformed human basal-like mammary epithelial MCF10A cells express high levels of ΔNp63 and low levels of MDM2 and BRCA1 relative to the lumenal MCF7 cells (compare Figure 5a, and Figure 6a). As inhibition of BRCA1 or MDM2 in lumenal cells resulted in induction of basal-like marker ΔNp63, we asked whether the opposite would be true for basal-like cells. We found that a siRNA-mediated knock-down of *TP63* in MCF10A cells increased the expression levels of MDM2 and BRCA1 3- and 2.5-fold, respectively, relative to a control siRNA (Figure 6b). Isogenic MCF10A cells with an engineered deletion of *TP53* (*TP53^−/−^* MCF10A) showed a lower level of ΔNp63 protein (Figure 6c), higher level of MDM2 (Figure 6d), BRCA1 (Figure 6e), TAp63 (Figure 5e), and lumenal cytokeratin KRT18 (Supplementary Figure S5a, b). Upregulation of KRT18 in *TP53^−/−^* MCF10A cells is likely mediated by TP63 as a knockdown of ΔNp63 led to a further increase in KRT18, while a knockdown of TAp63 resulted in a decrease of KRT18 and increase of the basal KRT5 (Supplementary Figure S5).

**Figure 7:**
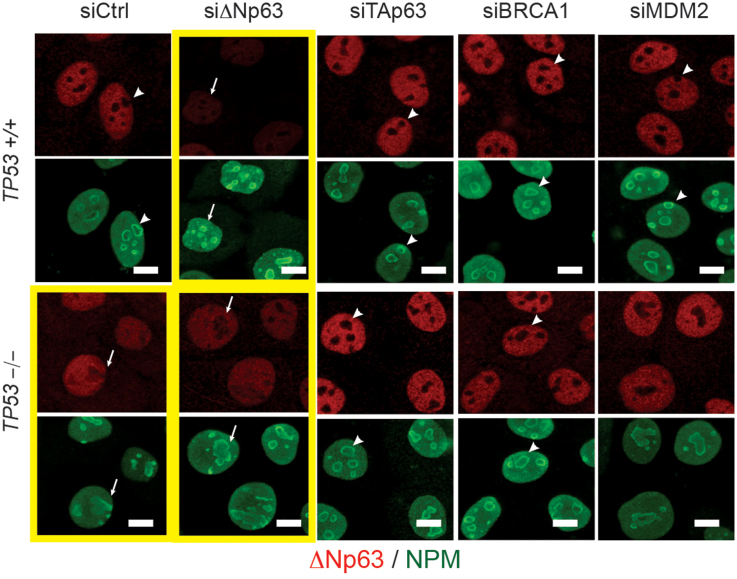
Inhibition of TAp63, BRCA1, or MDM2 in *TP53^−/−^* cells restores the nucleoplasmic localization of ΔNp63. Isogenic *TP53^+/+^* or *TP53^−/−^* MCF10A cells were co-stained for ΔNp63 (red) and NPM (nucleophosmin, green) to reveal their distribution relative to nucleoli after siRNA treatments indicated above. Yellow frames highlight a suppressed state of ΔNp63. Notice that in these cells ΔNp63 is found in nucleoli as well as in the nucleoplasm, and NPM is almost evenly distributed within nucleoli (arrows). Knockdown of TAp63 and others restores a nucleolar exclusion of ΔNp63 and a peripheral distribution of NPM within nucleoli (arrowheads).

We showed earlier that, in contrast to normal cuboidal MCF10A cells, a suppressed function of ΔNp63 in lumenal MCF7, basal EMT-like MDA-MB-231, and sparsely seeded basal MCF10A cells correlated with a nucleolar localization of the protein (Figure 3b, c, Figure 5a, and Figure 6a). A genetic deletion of *TP53* in MCF10A led to a relocation of the ΔNp63 protein from the nucleoplasm into nucleoli either completely (Figure 6e) or partially, filling out the entire nucleus including the nucleolar space (Figure 3c). Interestingly, the deletion of *TP53* and localization of ΔNp63 also affected the distribution of the nucleophosmin within nucleoli (Figure 3e, and Figure 7). In all cells showing a nucleolar localization of ΔNp63 (MCF7, MDA-MB-231, *TP53^−/−^* MCF10A, or siΔNp63 MCF10A) nucleophosmin is rather evenly distributed within nucleoli, while in *TP53^+/+^* MCF10A cells it is mostly found at the periphery of each nucleolus (Figure 3c, Figure 7). Nucleophosmin is an important nucleolar protein supporting translation of ribosomal proteins and associated with the nucleolar fibrillar component. Its localization depends on the functional state of nucleoli^28^. Although the connection between nuclear and nucleolar distribution of ΔNp63 and nucleophosmin is unclear, localization of nucleophosmin can serve as an additional indicator of TP53-related effects in MCF10A cells. Remarkably, while TAp63, MDM2, and BRCA1 were upregulated in *TP53^−/−^* MCF10A cells, a siRNA-mediated suppression of any of these genes restored both a nucleolus-excluded nucleoplasmic localization of ΔNp63 and a peripheral distribution of nucleophosmin within nucleoli (Figure 7). This suggests that these three induced genes are functionally relevant and their pharmacological targeting may help to treat TP53-deficient tumors by restoring the activity of ΔNp63 and the associated basal epithelial qualities.

## Discussion

In spite of various observations suggesting a cell type-specific role for *TP53* in epithelial tissues, particularly in mammary epithelia, no such role has been described for *in vivo* models of *Trp53*. Here we found that a loss of *Trp53* in mMECs has a negative effect on expression of basal-like features while having the opposite effect on the lumenal features, when a simple *in vitro* differentiation assay was used. The exact factors causing primary mMECs to change their phenotype from lumenal to basal, and from basal to mesenchymal in our assay are unclear. However, an epidermal growth factor (EGF) present in the medium is shown to promote the expansion of myoepithelial cells^29^. Adhesive culture conditions also support the expression of basal and inhibit lumenal cytokeratins^30^. Cells cultured on plastic in the absence of a basement membrane upregulate TGFβ1, which promotes EMT and stimulates MDM2 expression^31^,^32^. Thus, a combined effect of EGF and adhesive culture conditions are likely the key factors underlying the assay.

**Figure 8:**
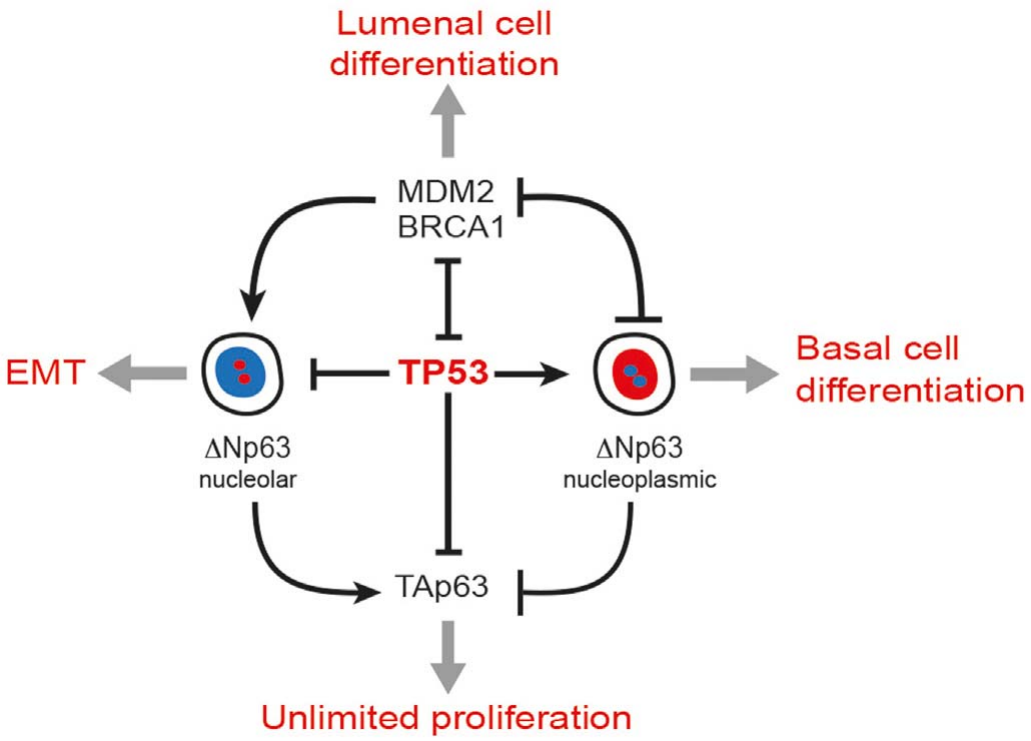
Model describing the role of TP53 in mammary epithelia. TP53 prevents translocation of ΔNp63 from the nucleoplasm into nucleoli. Active TP53 and a nucleoplasmic ΔNp63 inversely correlate with MDM2, BRCA1, and TAp63. MDM2 and BRCA1 in luminal cells inhibit TP53, thus preventing the nucleoplasmic expression of ΔNp63 and suppressing the basal-like differentiation. Loss of TP53 leads to an upregulation of TAp63, which is associated with an unlimited cell proliferation rather than a particular epithelial subtype. Relocation of ΔNp63 to nucleoli in basal epithelial cells is associated with EMT.

Our data suggest that a network of at least four genes maintain the cell type identity of mammary epithelial cells: TP53 and ΔNp63 are required for the basal subtype, while MDM2 and BRCA1 prevent their expression, thus, supporting lumenal cells (Figure 8). The key role of ΔNp63 in the maintenance of epidermal stem cells and basal epithelial cell types is well established, although mechanistic details are still being revealed^33^. However, this is the first report showing a direct role of TP53 in regulating ΔNp63. TP53 supports the overall expression level of the ΔNp63 protein as well as prevents its translocation from the nucleoplasm into nucleoli. As TP53 is a transcription factor, and validated TP53-binding sites are found in the promoter region of both isoforms of *TP63* (Supplementary Figure 1), the transcriptional regulation is likely to be direct. Future studies will elucidate mechanistic details of such regulation. A TP53-mediated nucleolar translocation of the ΔNp63 protein is also reported here for the first time. Although the underlying mechanism is unclear, nucleoli are known to play important roles in cell cycle control, stress responses, and aging^34^. Such spatial sequestration and the ubiquitin-mediated protein degradation belong to essential nucleolar functions^35^. Our data demonstrate that a nucleolar sequestration of the ΔNp63 protein is functionally equivalent to its inactivation using HDAC1/2 inhibitors and its knockdown leading to the upregulation of MDM2 and BRCA1. In turn, high level of MDM2 may suppress the basal-like epithelial differentiation by inhibiting ΔNp63 either via inactivation of TP53 or by directly interacting with ΔNp63 and promoting its nuclear export and proteasomal degradation mediated by the E3 ubiquitin ligase activity of FBXW7^36^. Changes in MDM2 expression correlated with those of BRCA1 in our experiments. A deletion of *BRCA1* in mammary progenitor cells prevents their lumenal differentiation, which may explain the bias towards a basal-like rather than lumenal phenotype for *BRCA1*-deficient breast cancers^37^. Our results offer an alternative explanation for this phenomenon. As an inactivation of *BRCA1* would likely lead to replication defects and DNA damage, it would elicit an activation of cell cycle checkpoints including the TP53 pathway, which could then promote the expression of ΔNp63 and basal-like differentiation.

In agreement with published reports, we show that a deletion of *Trp53* in mMECs eventually leads to EMT and an unlimited cell proliferation^32^,^38^. At the molecular level, this correlates with an upregulation of vimentin, MDM2, and TAp63, and a suppression of cytokeratins along with a nucleolar sequestration of ΔNp63. Once again, the transcription of TAp63 may be directly regulated by TP53 as several active binding sites for TP53 are identified in the promoter of TAp63, and an increase in TAp63 expression is independently confirmed in *TP53^−/−^* isogenic MCF10A cells. TAp63 can transactivate MDM2^39^, which, as discussed earlier, suppresses ΔNp63 leading to a loss of the epithelial differentiation. TAp63 can also promote EMT independently from ΔNp63 via the upregulation of transcription factors Slug and Snail^20^. As the TAp63 protein was found in nucleoli in all mammary epithelial cell lines irrespective of the histological subtype or EMT status, it may not be solely responsible for the EMT. Consistently, TAp63 has been linked to sustaining a long-term self-renewal capacity of epidermal progenitor cells^23^,^40^, thus adding to the transforming ability of a mutant TP53.

Taken together, our results demonstrate a role for TP53 in the EMT and differentiation of mammary epithelia, which is closely linked to the regulation of alternative isoforms of *TP63*. This role of *TP53* may explain the prevalence of sarcomas in *Trp53*-deficient animal models and Li-Fraumeni syndrome patients, and shed light on the mechanisms underlying observed differences in TP53 mutation spectra between lumenal and basal breast cancers.

## Methods

### Mice

B6.129S2-Trp53*^tm1Tyj^*/J mice carrying a deletion of exons 2–6 of *Trp53* were obtained from The Jackson Laboratory and maintained in a standard special pathogen free (SPF) facility at the University of Helsinki. The reporter mice were from Prof. Janet Sawicki laboratory from the Lankenau Institute for Medical Research^41^. All animal experiments were approved by the National Animal Experiment Board of Finland (Eläinkoelautakunta, ELLA) in compliance with the Finnish Act on Animal Experimentation (62/2006).

### Cell culture and drug treatments

MCF7, HCC1937, MDA-MB-231, and MCF10A cell lines were obtained from the American Type Culture Collection (ATCC). X-MAN isogenic *TP53^−/−^* and a matched parental MCF10A cells were purchased from the Horizon Discovery Inc. (cat # HD 101-005, and # HD PAR-024, respectively)^42^. Dulbecco's modified Eagle's medium (DMEM, Lonza) was used to maintain MCF7 and MDA-MB-231 cells. HCC1937 cells were cultured in RPMI 1640 (Lonza). Both media were Supplementaryemented with 10% fetal bovine serum (FBS), glutamine, penicillin, and streptomycin. Primary mMECs were isolated from inguinal mammary glands of adult virgin mice according to a protocol published elsewhere^43^. Isolated mMECs were cultured in standard tissue culture plastic plates in the DMEM/F12 growth medium without phenol red (Gibco) Supplementaryemented with 5% horse serum, 10 µg/ml insulin, 20 ng/ml EGF, 5 ng/ml choleratoxin, 500 ng/ml hydrocortison, penicillin, and streptomycin. MCF10A cells were cultured in the same medium. Cells were treated with Trichostatin A (Selleck) at 1–5 µM, or with XI-011/NSC 146109 (R&D) at 0.5 µM concentrations for 48 h. Treatments with Serdemetan (JNJ-26854165, Selleck) were carried out at 10 µM for 72 h.

### Immunofluorescence

Cells grown on glass coverslips were fixed with a 4% paraformaldehyde (PFA) for 15 minutes or 100% methanol for 10 minutes. Primary antibodies were used as follows: goat anti-TAp63 (Santa Cruz, sc-8608, diluted 1:50), rabbit anti-Ki67 (Abcam, ab15580, diluted 1:200), goat anti-ΔNp63 (Santa Cruz, sc-8609, diluted 1:100), rabbit anti-MDM2 (Abcam, ab58530, diluted 1:100), mouse monoclonal anti-Krt18 (Abcam, ab668, diluted 1:100), mouse monoclonal anti-p53 (Abcam, ab26, diluted 1:100), mouse monoclonal anti-p53 (Abcam, PAb 240, diluted 1:100), mouse anti-human BRCA1 (Calbiochem, OP92, diluted 1:100), and mouse monoclonal anti-Nucleophosmin (Invitrogen, FC-61991, diluted 1:200, a kind gift of Karita Peltonen and Prof. Marikki Laiho). Secondary antibodies were donkey anti-goat IgG conjugated with Texas Red (Invitrogen, diluted 1:1000), or goat anti-rabbit IgG conjugated with Alexa Fluor 594 (Invitrogen, diluted 1:1000), or goat anti-mouse IgG conjugated with Alexa Fluor 488 (Invitrogen, diluted 1:1000). Images were taken with a Nikon Eclipse 90i fluorescence microscope and processed using Nikon NIS-Elements AR software.

### Gene Silencing and expression analysis

Transient gene-specific knockdowns were carried out using following siRNAs (Qiagen): siMDM2 (cat. # SI02653392, target sequence 5′-AACCTGAAATTTATTCACATA-3′), siBRCA1 (cat. # SI02654575, target sequence 5′-CAGCAGTTTATTACTCACTAA-3′), siTP63 (cat. # SI04951443, target sequence 5′-AACCATGAGCTGAGCCGTGAA-3′), siTP53 (cat. # SI02655170, target sequence 5′-AAGGAAATTTGCGTGTGGAGT-3′). Custom siRNAs against TAp63 (target sequence 5′-GCGACAAACAAGATTGAGA-3′) or ΔNp63 (target sequence 5′-TGCCCAGACTCAATTTAGT-3′) were ordered from Sigma. siRNA duplexes at a final concentration 20 nM were transfected using Lipofectamine RNAiMAX (Invitrogen) according to manufacturer's instructions. Total RNA was extracted using the NucleoSpin RNA II kit (Macherey-Nagel) 48 hours after siRNA transfection. cDNA was synthesized using Revert Aid First Strand cDNA Synthesis Kit (MBI-Fermentas). Gene expression was measured by real-time quantitative PCR (qRT-PCR) using the SYBR-Green-based detection method and CFX384 cycler (Bio-Rad). Primer sequences are listed in Supplementary Table 1. Gene expression was normalized relative to GAPDH and quantified using the CFX software (Bio-Rad).

### Western blotting

Cells were lysed in a RIPA buffer. 15 μg proteins were separated using NuPAGE 4–12% Bis-Tris, precast gels (Invitrogen) according to manufacturer's instructions, and transferred to the Immobilon-FL PVDF membrane (Millipore). Membranes were incubated with primary antibodies in a blocking buffer (1xTBS, 0.05% Tween 20, 5% Milk) overnight at +4°C. After incubation with fluorescently labeled secondary antibodies (goat-anti-mouse IRDye 800 CW or goat-anti-rabbit IRDye 680 LT, LI-COR) diluted 1:10000, membranes were scanned using a fluorescence scanner Odyssey (LI-COR Biosciences).

### Chromatin immunoprecipitation followed by quantitative PCR (ChIP-qPCR)

ChIP assay was performed as previously described^44^. Briefly, MCF10A cells were cross-linked with 1% formaldehyde and quenched with 125 mM glycine. Nuclei were isolated and lysed in SDS lysis buffer (50 mM Tris-HCl, pH 8.1, with 0.5% SDS, 10 mM EDTA and Complete Protease Inhibitor cocktail). Nuclear extracts were sonicated to generate chromatin fragment with an average size of 0.6 kb using a Q800R sonicator (QSonica). Chromatin lysate was cleared by centrifugation and diluted with 6 volumes of cold IP buffer (EDTA 2 mM, NaCl 150 mM, Tris-HCl 20 mM, pH 8.0, Triton X-100 1%, Complete Protease Inhibitor), and incubated overnight at 4°C with the Dynabead/antibody complex (80 ul Dynabead protein G (Invitrogen) contained 8 μg antibody against TP53 (sc-126, Santa Cruz Biotechnology) or control IgG (normal mouse IgG, sc-2025, Santa Cruz Biotechnology) in Blocking buffer (0.5% BSA in IP buffer). After washing, the protein-DNA complex was eluted with 100 μl extraction buffer (1 mM EDTA, 1% SDS in 10 mM Tris-HCl, pH 8.0), and treated with Proteinase K (1 mg/ml) and NaCl (0.3 M) overnight at 65°C. Then RNase A (0.2 mg/ml) was added for 1 h at 37°C. DNA was purified with the Mini-Elute PCR purification kit (Qiagen) and analyzed by qPCR with EpiTect ChIP qPCR Primer set for TP63 (cat # GPH1009765(+)03A, Qiagen). A relative enrichment of the target DNA fragments was assessed by calculating the immunoprecipitation efficiency above fragment-specific background (IgG control) followed by normalization to the occupancy level observed for the control region amplified with primers ChIPneg135f (5′- TGCCTCAGATTTGGAGTGCT-3′) and ChIPneg135r (5′- GAGAAGCCTCTGAGGAGGGA-3′).

## Author Contributions

P.M.M. and S.G.K. designed the experiments, analyzed the data, and wrote the main manuscript text. J.S. provided the reporter mice. P.M.M. isolated and cultured primary cells. Y.G. performed most Western blotting experiments. P.M.M., M.T. and Y.G. carried out the qRT-PCR experiments. P.M.M., S.K. and S.U. performed the immunofluorescence stainings. P.G. and G.-H.W. performed the ChIP qPCR experiments. S.G.K. supervised the project. All authors reviewed the manuscript.

## Supplementary Material

Supplementary Information Supplementary Information
