# Supplementary material for: TP53 supports basal-like differentiation of mammary epithelial cells by preventing translocation of deltaNp63 into nucleoli
Source: Sci Rep. 2014 Apr 11;4:4663. doi: 10.1038/srep04663 (PMC3983616; doi:10.1038/srep04663)
Supplement: Supplementary Information [file srep04663-s1.pdf]

TP53 supports basal-like differentiation of mammary epithelial cells by preventing translocation of ΔNp63 into nucleoli

Pauliina M. Munne, Yuexi Gu, Manuela Tumiati, Ping Gao, Sonja Koopal, Sanna Uusivirta, Janet Sawicki, Gong-Hong Wei & Sergey G. Kuznetsov

SUPPLEMENTARY FIGURE LEGENDS

Supplementary Figure S1. TP53 binds in the promoter region of *TP63*. (a) Functionally validated binding sites for TP53 are found in promoters of both isoforms of TP63 (red arrows) suggesting an active regulatory role. A genomic locus of the human chromosome 3 within given genetic coordinates shows a promoter area for TP63. ChIP-Seq-validated binding sites for transcription factors are shown as green vertical lines. The data are obtained from The Champion ChiP Transcription Factor Search Portal (Qiagen, http://www.sabiosciences.com/chipqpcrsearch.php?app=TFBS). (**b**) ChIP qPCR assay performed on MCF10A cells to confirm binding of TP53 within the promoter region of TP63. Chromatin was precipitated with IgG or TP53-specific antibodies and amplified with primers flanking the TP53-binding site marked with red in (**a**).

Supplementary Figure S2. Lumenal mMECs transdifferentiate into basal cells when cultured in adhesive conditions. Primary wild type mMECs isolated from mice carrying both lumenal K18-RFP and basal K5-CFP reporters reveal a gradual loss of both keratins when cultured on plastic (upper panel), while maintaining their expression when cultured in matrigel (lower panel). RFP, red fluorescent protein; CFP, cyan fluorescent protein. Scale bars correspond to 50 μm.

Supplementary Figure S3. TP53 is weakly expressed in untreated lumenal MCF7 cells (**a**), but can be stabilized using a pharmacological treatment with a MDM4 inhibitor XI-001 (**b**), or by incubating in a basal cell-supporting medium DMEM/F12 (**c**). Scale bars correspond to 20 μm.

Supplementary Figure S4. DMEM/F12 medium forces lumenal MCF7 cells to express basal markers, which can be reversed by knockdown of TP53. siRNA specificities are shown above. Antibody stainings are indicated on the left. Long

arrows show a nucleolar localization of ΔNp63. A short arrow indicates ΔNp63 protein in the nucleoplasm. Scale bars correspond to 20 μm.

Supplementary Figure S5. Combination of TP53, ΔNp63, and TAp63 regulate expression of lineage-specific keratins in MCF10A cells. (a) Western blot showing the effect of TP53 deletion and knockdown of ΔNp63 on the lumenal marker KRT18. (b) Western blot demonstrating opposite effects of ΔNp63 and TAp63 depletion on lumenal KRT18. (c) Depletion of TAp63 in *TP53*$^{-/-}$ MCF10A cells results in a higher expression of basal KRT5, while TSA treatment of parental *TP53*$^{+/+}$ MCF10A cells associated with inhibition of ΔNp63 leads to a reduced expression of KRT5. TSA, trichostatin A; GAPDH, Glyceraldehyde-3-Phosphate Dehydrogenase; MW, molecular weight marker.

Munne_Suppl Figure S1.

a

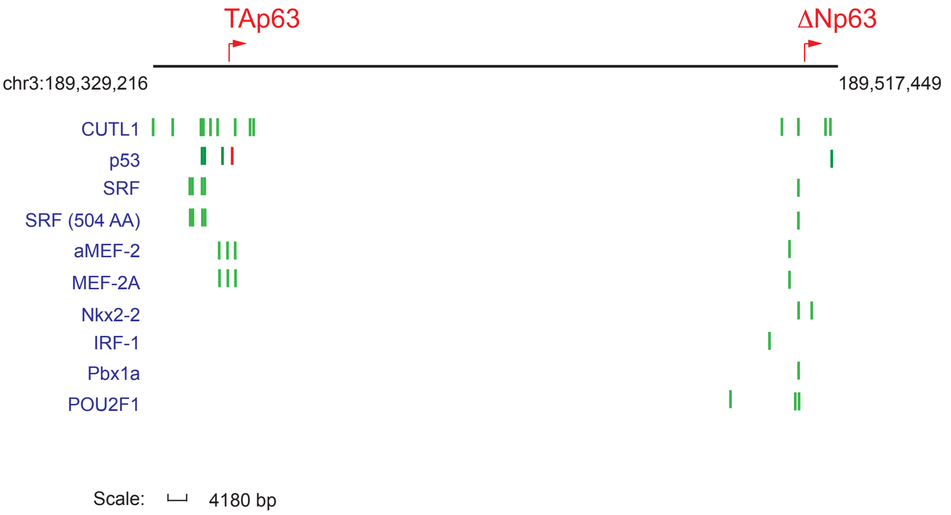

b

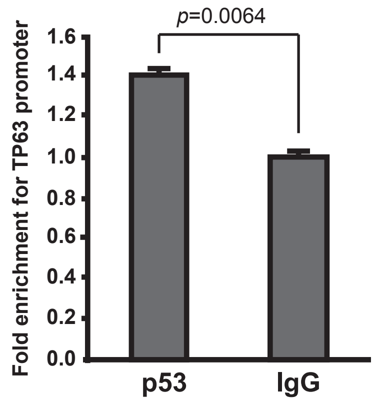

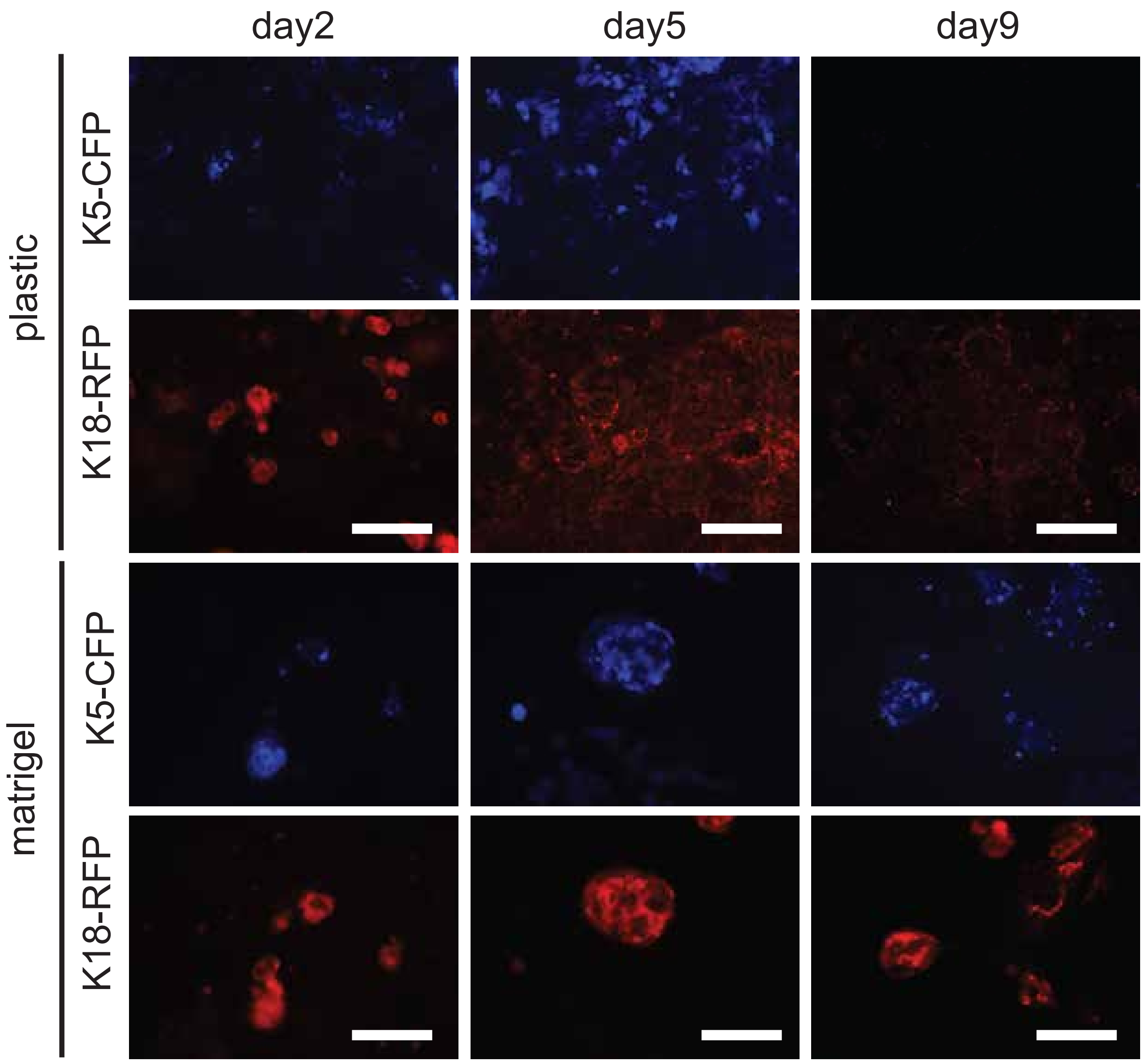
day2
day5
day9
plastic
K5-CFP
K18-RFP
matrigel
K5-CFP
K18-RFP

Supplementary Figure S3.

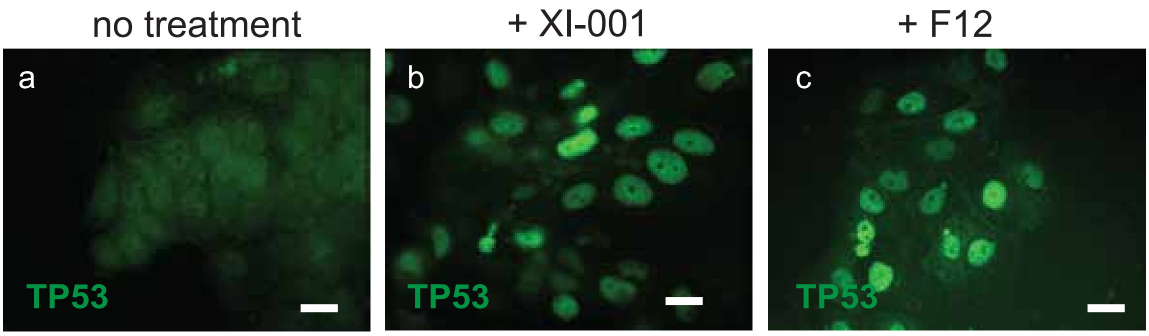

Munne_Suppl Figure S4.

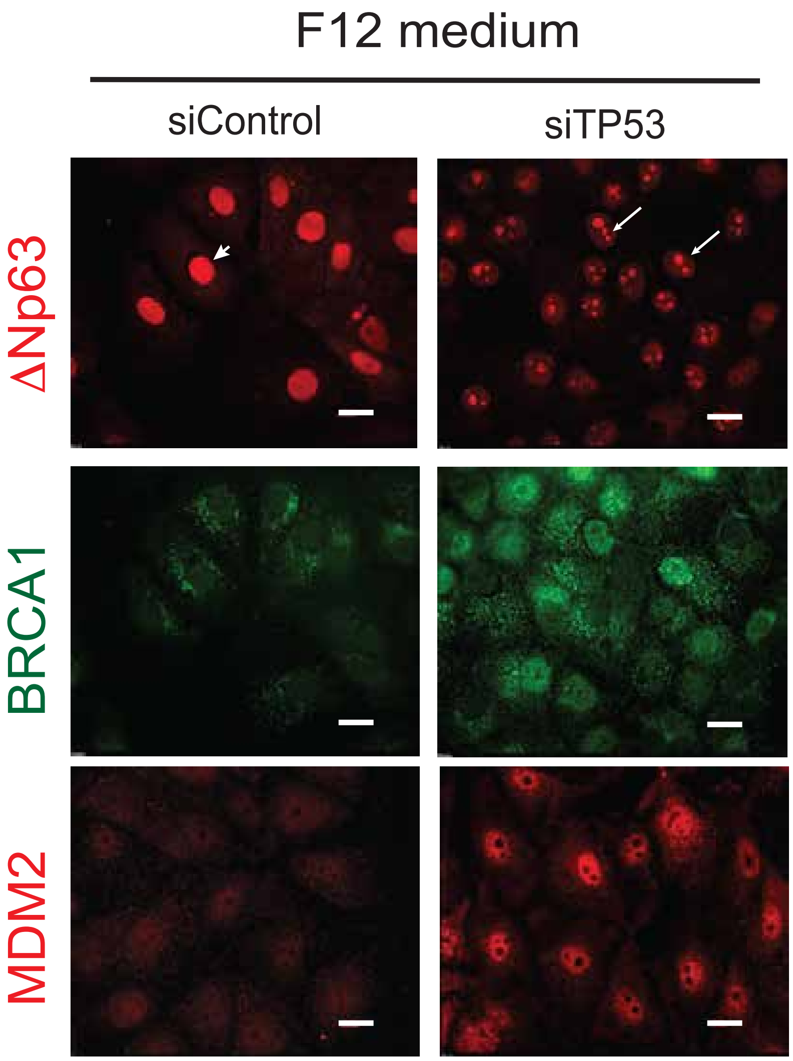

Munne_Suppl Figure S5.

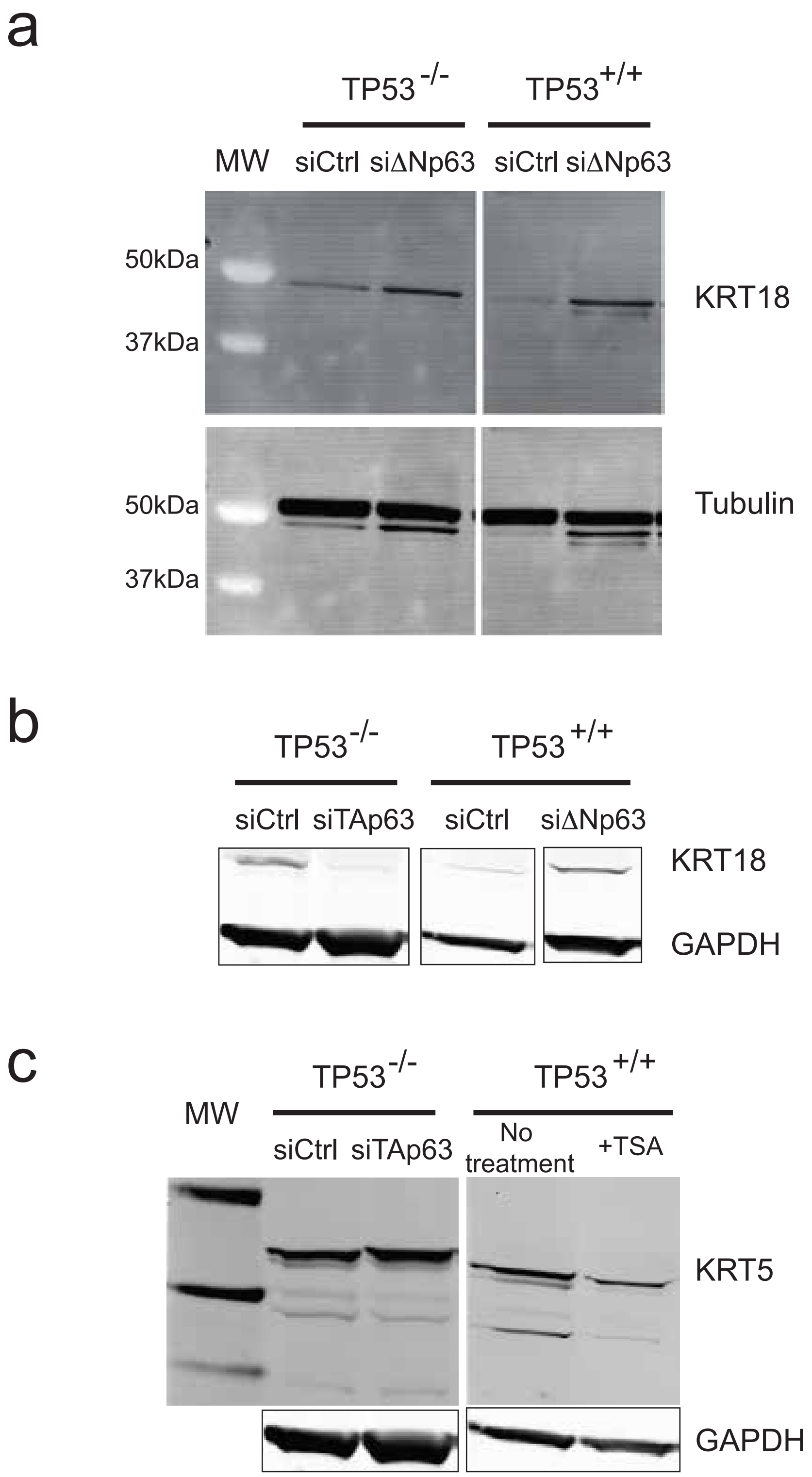

Supplementary Table 1. Sequences of qPCR primers

Species	*Target gene*	*Sense primer*	*Antisense primer*
Mouse	Gapdh	AATGGTGAAGGTCGGTGTG	CTGGAAGATGGTGATGGGC
Mouse	Brca1	CAAGGCGAGAGCTAGAAGGA	GGAAAGCAACTTGACCTTGG
Mouse	Mdm2	CTCTGGACTCGGAAGATTACAGCC	CCTGTCTGATAGACTGTGACCCG
Mouse	TAp63	GTGGATGAACCTTCCGAAAA	GAGGAGCCGTTCTGAATCTG
Mouse	ΔNp63	CAAAACCCTGGAAGCAGAAA	GAGGAGCCGTTCTGAATCTG
Mouse	Vimentin	GACCTTGAACGGAAAGTGGA	AGCCACGCTTTCATACTGCT
Human	GAPDH	ACGGGAAGCTTGTCATCAAT	TTCAGCTCAGGGATGACCTT
Human	BRCA1	ACAAATACTCATGCCAGCTCAT	GGCTCCTTGCTAAGCCAGG
Human	MDM2	CTGGCTCTGTGTGTAATAAGGGAG	CCTGATCCAACCAATCACCTG
Human	TAp63	TGTATCCGCATGCAGGACT	CTGTGTTATAGGGACTGGTGGAC
Human	ΔNp63	GAAAACAATGCCCAGACTCAA	TGCGCGTGGTCTGTGTT
